# Neuroprotective Strategies for Retinal Ganglion Cell Degeneration: Current Status and Challenges Ahead

**DOI:** 10.3390/ijms21072262

**Published:** 2020-03-25

**Authors:** Raquel Boia, Noelia Ruzafa, Inês Dinis Aires, Xandra Pereiro, António Francisco Ambrósio, Elena Vecino, Ana Raquel Santiago

**Affiliations:** 1 Coimbra Institute for Clinical and Biomedical Research (iCBR), Faculty of Medicine, University of Coimbra, 3000-548 Coimbra, Portugal; 2 Center for Innovative Biomedicine and Biotechnology (CIBB), University of Coimbra, 3000-548 Coimbra, Portugal; 3 Department of Cell Biology and Histology, University of the Basque Country UPV/EHU, Leioa 48940, Vizcaya, Spain; 4 Association for Innovation and Biomedical Research on Light and Image (AIBILI), 3000-548 Coimbra, Portugal

**Keywords:** retinal ganglion cells, neurodegeneration, axonal regeneration, neuroprotection, optic neuropathies

## Abstract

The retinal ganglion cells (RGCs) are the output cells of the retina into the brain. In mammals, these cells are not able to regenerate their axons after optic nerve injury, leaving the patients with optic neuropathies with permanent visual loss. An effective RGCs-directed therapy could provide a beneficial effect to prevent the progression of the disease. Axonal injury leads to the functional loss of RGCs and subsequently induces neuronal death, and axonal regeneration would be essential to restore the neuronal connectivity, and to reestablish the function of the visual system. The manipulation of several intrinsic and extrinsic factors has been proposed in order to stimulate axonal regeneration and functional repairing of axonal connections in the visual pathway. However, there is a missing point in the process since, until now, there is no therapeutic strategy directed to promote axonal regeneration of RGCs as a therapeutic approach for optic neuropathies.

## 1. Introduction

**Figure 1:**
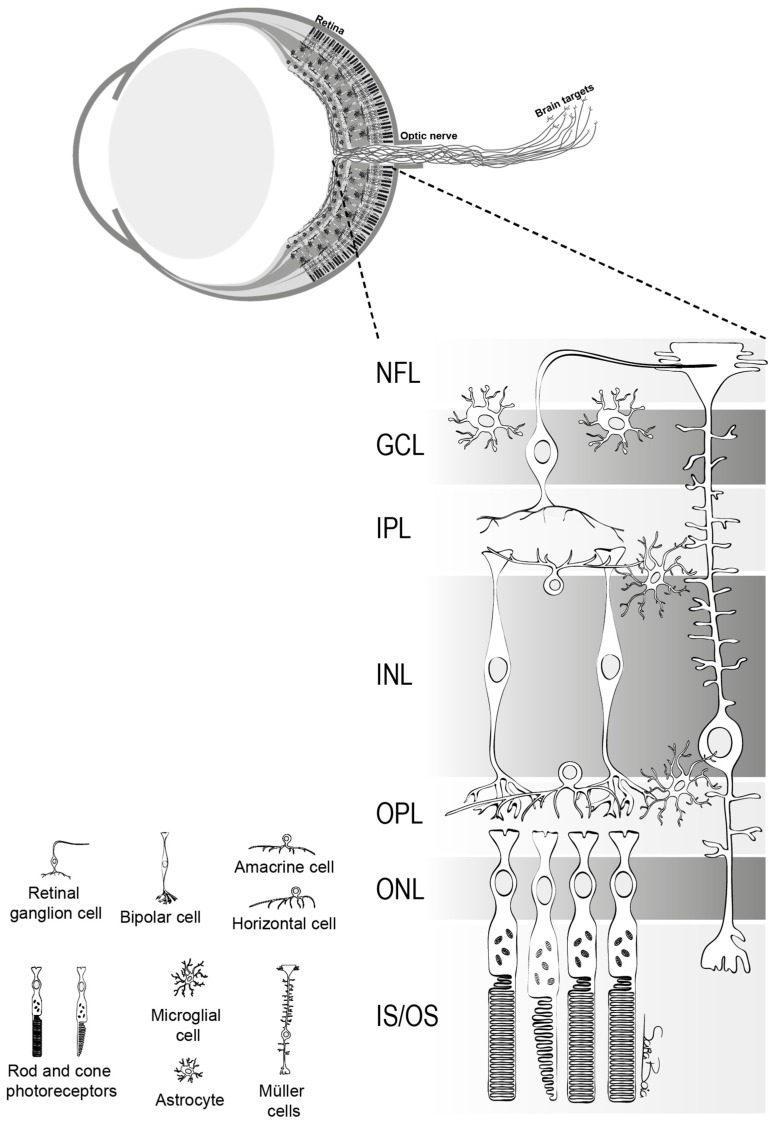
Schematic representation of the neural sensory retina, depicting the organization of the cells into nuclear and plexiform layers. The nuclei of photoreceptors, rods and cones, are located in the outer nuclear layer (ONL) and nuclei of interneurons, amacrine, bipolar and horizontal cells, are located predominately in the inner nuclear layer (INL). The cell bodies of RGCs are in the ganglion cell layer (GCL), and their axons run in the nerve fiber layer (NFL). There are two types of macroglia: Müller cells that span vertically the entire retina and astrocytes that are present in the GCL. Microglial cells are localized predominately in the inner retina and in the outer plexiform layer (OPL). IPL: inner plexiform layer; IS/OS: inner and outer segments of photoreceptors.

The retina is part of the central nervous system (CNS) and is constituted by neurons, glial cells and blood vessels [1]. The neuronal component of the retina is composed by six types of neurons: photoreceptors (rods and cones), bipolar cells, horizontal cells, amacrine cells and retinal ganglion cells (RGCs). Photoreceptors, whose nuclei is located in the outer nuclear layer (ONL), respond to light and make synapses with second-order neurons. The cell bodies of retinal interneurons (horizontal, bipolar and amacrine cells) are located predominately in the inner nuclear layer (INL) and modify and relay the visual information from the photoreceptors to the RGCs that are located in the innermost layer of the retina, the ganglion cell layer (GCL) (Figure 1). RGCs are the output cells of the retina that convey the visual signals to the brain visual targets. The axons of RGCs run initially in the nerve fiber layer (NFL) and converge into the optic disc, cross the lamina cribrosa at the optic nerve head (ONH), and form the optic nerve (Figure 1) [1].

Optic neuropathies comprise a group of ocular diseases, like glaucoma (the most common), anterior ischemic optic neuropathy and retinal ischemia, in which RGCs are the main affected cells [2]. Blindness secondary to optic neuropathies is irreversible since RGCs lack the capacity for self-renewal and have a limited ability for self-repair [3]. The exact mechanism that leads to RGC death and degeneration is still unknown, but axonal injury has been proposed as an early event that culminates in apoptotic death of RGCs [4]. This paper reviews the events that contribute to axonal degeneration and death of RGCs and also the neuroprotective strategies with potential to circumvent this problem.

## 2. Obstacles to RGC Survival and Regeneration upon Injury: Insights from Development to Disease Models

**Figure 2:**
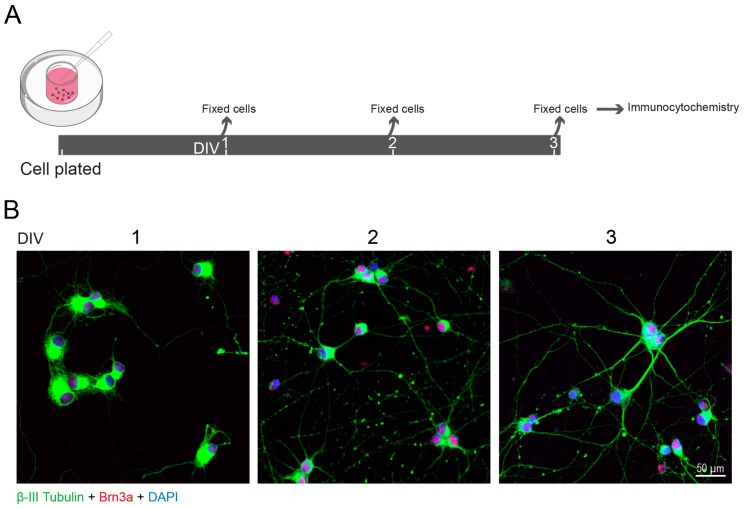
Neurite growth of RGCs in culture. (**A**) Schematic representation of the experimental design. Retinas were dissected from Wistar rats at PND 5 and nearly pure RGC cultures (~93% purity assessed with anti-RBPMS antibody; Abcam, Cat. # ab194213, 1:500) were obtained by sequential immunopanning, as previously described [8,9]. RGCs were cultured for 1 day in vitro (DIV1), DIV2 and DIV3, followed by fixation in paraformaldehyde and processed for immunocytochemistry. (**B**) RGCs were identified by immunolabeling for Brn3a (red, Millipore, Cat. # MAB1585, 1:500), a transcription factor expressed only by these cells in the retina. The neurites, labelled with an antibody that recognizes β-tubulin III (green, BioLegend, Cat. # 802001; 1:1000), extended during the period in culture. Nuclei were stained with DAPI (blue).

During development, RGCs extend their axons to synapse in target areas of the brain (reviewed in [5]). After birth, there is a peak in cell death that in rodents occurs between postnatal days 2 and 5 (PND 2-5), ensuring that only cells that reached their targets survive (reviewed in [6]). The ability of RGCs to extend their axons decreases with age and the capacity to regenerate their axons is lost early in development [7]. In fact, cultures of RGCs (Figure 2) prepared at both embryonic day 20 (ED 20) or PND 8 extend their axons with similar calibers; however, after 3 days in culture, ED 20 RGCs extend their axons further and faster than cells isolated at PND 8. The exposure of these cells to conditioned media of superior colliculus cells further potentiates axonal growth of ED 20 RGCs without interfering with PND 8 RGCs, demonstrating that the loss of ability of RGCs axon growth is mediated by retinal maturation [7]. The reason behind the lost in the intrinsic ability of RGCs to regenerate upon injury has been extensively explored. Several players, including cyclic adenosine monophosphate (cAMP), phosphatase and tensin homologue (PTEN)/mammalian target of rapamycin (mTOR) and Krüppel-like family (KLF) transcript factors are implicated in the transition from the rapid axon growth of immature neurons into the poor axon growth of mature neurons in the CNS.

cAMP plays an important role in neuronal survival and axon growth and guidance [10]. For example, in the goldfish, the injection of an analogue of cAMP is able to enhance axonal regeneration upon optic nerve crush (ONC) [11]. Moreover, PTEN/mTOR pathway has been implicated in the failure of RGCs axons to regenerate. The deletion of PTEN in RGCs leads to the activation of phosphoinositide 3-kinases (PI3K)/mTOR pathway, increases neuronal survival and promotes robust axon regeneration after optic nerve injury [12,13]. Moreover, it has been reported a coordinated regulation of neurite growth by KLF transcription factors. During development, at least two growth-enhancing KLFs (KLF6 and 7) are down-regulated, and at least two growth-suppressive KLFs (KLF4 and 9) are upregulated [14]. The profile of gene expression from ED 17 through PND 21 RGCs identified the zinc finger transcription factor KLF4 as the most effective suppressor of neurite outgrowth [14]. Indeed, the KLF4 overexpression in ED 20 RGCs reduces their ability to extend axons and, on the other hand, KLF4 knockout enhances axon growth ability by PND 12 RGCs [14]. This decline in the ability of postnatal RGCs to grow axons is associated with KLF-regulated changes in axonal growth cone morphology and protrusive dynamics [15]. The knockout of KLF4 during development increases the regenerative potential of RGCs upon ONC at adulthood [14]. Amacrine cells have been implicated in the process of losing intrinsic growth capability of RGCs [7]. In fact, zinc (Zn^2+^) increases in amacrine cell processes upon optic nerve injury and is transferred to RGCs via vesicular release [16]. The chelation of Zn^2+^ improves cell survival and axon regeneration [16], raising the possibility that the dysregulation of mobile Zn^2+^ levels is responsible for the loss of axonal growth.

Other transcription factors have been studied for their role in axon growth and regeneration (reviewed in [17]). The tumor suppressor p53 plays a central role in the regulation apoptosis in RGCs. The overstimulation of N-methyl-D-aspartate (NMDA) receptor activates a p53-dependent pathway of cell death [18]. The involvement of p53 in neurite outgrowth and axon regeneration has been explored in CNS injury [19]. However, the deletion of p53 in RGCs fails to promote axonal regeneration, despite the increase in RGC survival upon ONC [12], confirming the hypothesis that inducing neuronal survival is not enough to allow axonal regeneration. The activation of p53 has been implicated in the transcription of several factors responsible for apoptosis, as pro-apoptotic BAX or anti-apoptotic Bcl-2 proteins (reviewed in [20]). It was shown that there is an up-regulation of BAX expression after ONC injury [21], as well as after ischemic retinal damage [22]. BAX deficiency completely prevents RGCs death in a glaucoma animal model [23]. However, deficient BAX expression in not sufficient to hinder axonal degeneration even without RGC death, reinforcing the idea that axon degeneration is not a consequence of RGC death [23]. A down-regulation of the anti-apoptotic protein Bcl-2 was observed in RGCs in the GCL when the onset of regenerative failure of RGCs occurs [24]. Elevating the expression of Bcl-2 maintains neuronal survival even after withdrawing of all trophic factors in cultures of RGCs [3]. However, Bcl-2-overexpressing RGCs fail to elaborate axons or dendrites, unless axon growth-inducing signals are present, clearly demonstrating that axon growth is not a default function of a surviving neuron, but must be specifically signaled [3]. These evidences clearly demonstrate that manipulation of some intrinsic factors could have beneficial effects, not only in the prevention of RGC death but also in promoting axon regeneration upon injury. In the peripheral nervous system (PNS) the injured neurons are able to regenerate, which does not happen in the CNS. However, the observation that CNS neurons, including RGCs, regrow into peripheral nerve grafts [25,26], confirms the possibility that extrinsic factors also have a preponderant role in limiting axonal repair.

Glial scar and myelin that compose the environment of optic nerve particularly at the site of injury inhibit the axonal regeneration (reviewed in [27]). Semaphorin-3 is expressed in the core of the glial scar upon CNS injury [28] and limits regenerating neurons crossing semaphorin-3A (Sema3A)-expressing regions [29]. This raises the hypothesis that semaphorins may have a potential role in the glia inhibiting effect of axonal regeneration. Semaphorins have an important function in neuronal polarity and axonal guidance during RGC development or injury [30]. Sema3A is one of the extracellular factors that is involved in regulating RGC polarity [31,32]. At PND 14, when all RGCs axons reached their targets [33], Sema3A is elevated [34], and increased expression of Sema3A results in strong axonal inhibition in optic nerve injury model [35]. In line with these findings and corroborating the role of semaphorin in axonal growth, the intravitreous injection of antibodies against the Sema3A-derived peptide to neutralize the function of Sema3A, caused a marked inhibition of RGC loss in an animal model of complete axotomy of the rat optic nerve [36]. Sema5A is a semaphorin produced by oligodendrocytes that also contributes to the inhibitory environment of the injured optic nerve, heralded by the observation that RGC axonal growth increases when blocking Sema5A [37]. It has been demonstrated that myelin proteins inhibit axonal regeneration in adult neurons. Following an insult, nonspecific T cells accumulate at the lesion site on optic nerve [38,39]. Immunization with T cells specifically against myelin proteins (copolymer-1, Cop-1) reduces the post-traumatic neuronal loss after ONC [38,39]. Moreover, it has been shown to be an effective therapy for glutamate-induced toxicity in mice and in a rat model of chronically high intraocular pressure (IOP) [40]. Although these studies were only focused on the survival of RGCs, some years after the authors demonstrated that Cop-1 treatment confer functional protection to RGCs [41]. Other studies led to the identification of several myelin-associated inhibitors of axon growth. Nogo-A is one of the most potent oligodendrocyte-derived inhibitors for axonal regrowth in the injured adult CNS [42,43] that is also expressed by RGCs [44]. In cases of optic nerve injury Nogo-A is upregulated [45], although the overexpression or down-regulation of Nogo-A does not impact the survival of injured RGCs. However, the neuronal knockout of Nogo-A diminishes the axonal growth response, demonstrating a role for Nogo-A in RGCs growth after injury [45]. On the other hand, axonal sprouting is increased in the optic nerves of oligodendrocyte-specific Nogo-A knockout mice [46], demonstrating that the inactivation of Nogo-A in oligodendrocytes appears to be a good strategy to promote axonal regeneration. Moreover, it was reported that neutralizing Nogo-A has beneficial effects on visual recovery and plasticity after retinal injury [47]. Moreover, myelin-associated glycoprotein (MAG) is a component of the myelin-derived inhibition of nerve regeneration [48]. It seems that a possible mechanism underlying synapse degeneration and RGCs death in glaucoma is mediated by Nogo-A [49]. The antagonism of Nogo receptor (NgR) reduces RGCs loss and attenuates synaptic degeneration [50] and the knockout of NgR is effective in enhancing axonal regeneration after ONC [51].

The failure to regenerate has also been attributed to an environment poor in growth-promoting trophic factors. In fact, the importance of trophic factors in promoting viability and axonal regeneration of RGCs has long been recognized [51]. A great variety of neurotrophins were found to induce axon growth, which include nerve growth factor (NGF), brain-derived neurotrophic factor (BDNF) and ciliary neurotrophic factor (CNTF). BDNF plays an important role in RGCs neuroprotection since the levels of BDNF are increased in response to injury [52,53]. BDNF is also highly expressed in the superior colliculus [54,55] and it is retrogradely transported to the retina. However, displaced amacrine cells in the GCL are the main source of BDNF to RGCs [56]. The application of BDNF to the superior colliculus reduces RGC death during development [57]. Moreover, several studies demonstrated that administration of BDNF into the eye increases the survival of RGCs upon injury, and ameliorate their function [58,59,60,61,62,63,64].

The survival of RGCs is increased by co-administration of BDNF and CNTF soon after optic nerve injury [65]. Moreover, RGCs extend their axons in response to BDNF and CNTF, but both together induce more axon growth than either alone [3], raising the hypothesis that different factors may be responsible for different facets of axon growth. However, neurotrophins fail to induce axon growth alone. For instance, RGCs fail to survive in the presence of such trophic factors as BDNF or CNTF unless their cAMP levels are elevated [66]. CNTF overexpression promotes long-term survival and regeneration of injured adult RGCs [67]. It was described that exogenously applied CNTF stimulates RGCs partially indirectly via a mechanism that depends on astrocyte-derived CNTF [68]. The NGF has also an important role in promoting RGCs survival, being the Schwann cells the main source of this factor [69]. Intraocular injection of NGF has been previously shown to promote RGC survival [70].

**Figure 3:**
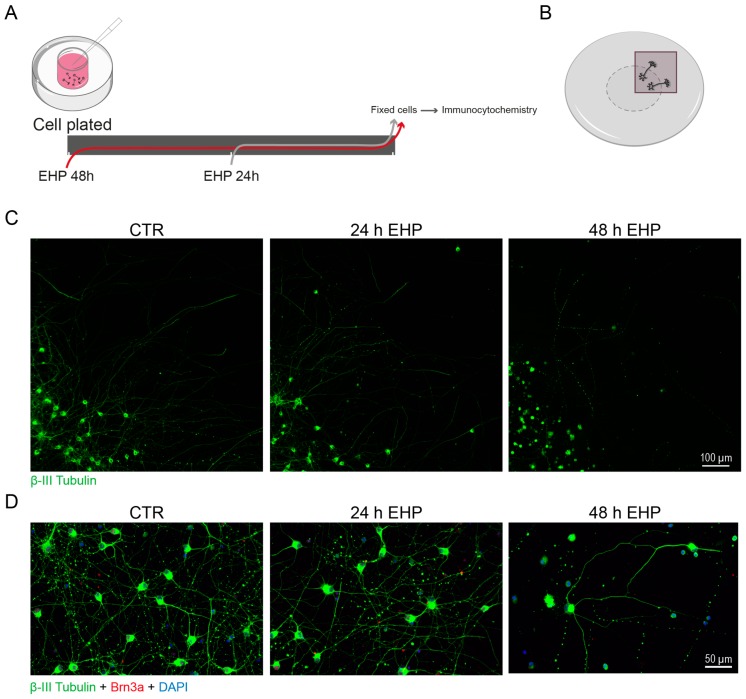
Elevated hydrostatic pressure (EHP) impacts neurite growth of RGCs. (**A**) Schematic representation of the experimental design. RGCs were purified from Wistar rats at PND 5 by sequential immunopanning, as previously described [8,9] and were cultured for DIV2. RGCs were challenged with EHP (+70 mmHg above atmospheric pressure) [78,79]) for 24 h and 48 h and then processed for immunocytochemistry as described in the legend of Figure 2. (**C**) RGCs were plated in a coverslip with a cloning cylinder and neurite extension was observed beyond the limit established by the cylinder (**B**, grey dashed circle). Exposure to EHP decreased the length of the neurites when compared with the control (CTR) condition (normal pressure). (**D**) Higher magnification. This effect on the neurites of RGCs is dependent on the duration of the exposure to EHP.

Studying the mechanisms of glaucomatous damage has been a great opportunity to unravel the signaling pathways involved in RGC axonal degeneration and growth. Elevated IOP is the main risk factor of glaucoma and, together with other factors, it has been implicated in RGC degeneration and death [71]. Several in vitro models have been developed [72] and allowed the demonstration that there are pressure-dependent changes in the length of axons and neurites of RGCs [73]. When cultures of RGCs are challenged with elevated pressure there is a severe impact in axon length and in the total neurite length, with a weakened neurite extension (Figure 3), without interfering with cell body area [73]. In glaucoma, the increased IOP perturbs anterograde and retrograde axonal transports that lead to deprivation of RGCs of neurotrophic factors produced by brain targets [74]. In fact, the retrograde transport of BDNF is impaired after IOP elevation, and this may contribute to RGC loss [75,76].

Recently, it was reported that intravitreal injections of BDNF leads to an increase in the levels of synaptic proteins between RGCs and bipolar cells in the IPL, meaning that this could have a beneficial effect in the function of RGCs [77].

## 3. Potential Therapeutic Targets Aiming RGC Neuroprotection

**Figure 4:**
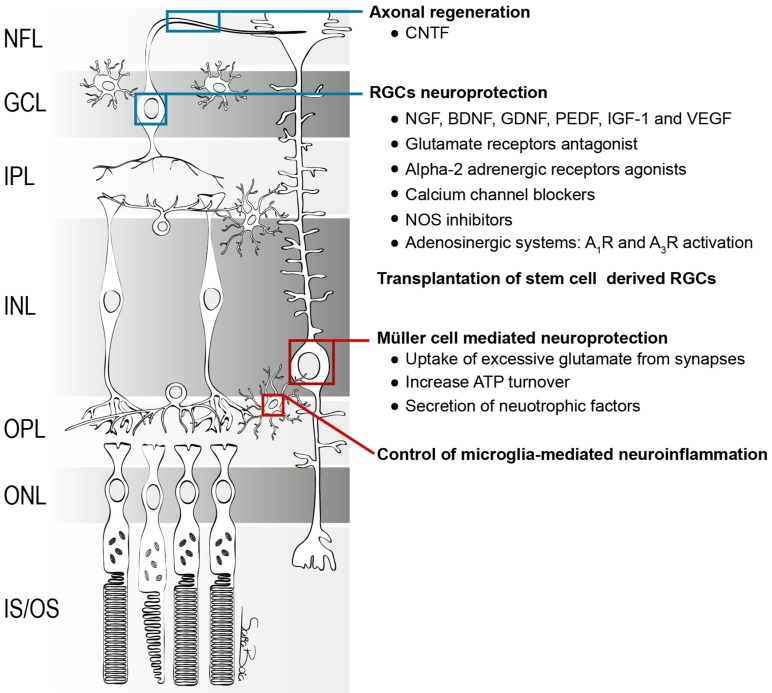
Schematic representation of the main strategies for RGC neuroprotection. Blue squares represent the therapies directed to RGCs and red squares represents the undirected therapies that culminates in RGCs neuroprotection.

Several therapeutic strategies have been proposed in order to protect RGCs and restore visual function (Figure 4).

### 3.1. Neuroprotective Therapies

#### 3.1.1. Neurotrophic Factors

Neurotrophic factors are a family of growth factors that regulate the survival, development and differentiation of neurons. Neurotrophic factors generally include the neurotrophin family: NGF, BDNF, neurotrophin-3 (NT-3) and neurotrophin-4/5 (NT-4/5); the glial cell-line derived neurotrophic factor (GDNF) family: GDNF, neurturin (NRTN), artemin (ARTN), and persephin (PSPN); and CNTF [80,81]. It was reported that most of these neurotrophic factors, which can be produced by glial cells, increase RGC survival in different experimental models of injury [53,82,83,84,85,86,87,88]. Neurotrophic factors bind to different receptors and transduce diverse intracellular signals. Usually, neurotrophic factors bind to the high affinity receptor tyrosine kinase (Trk family) that promote cell survival. For instance, NGF binds to TrkA, BDNF and NT-4 to TrkB, and NT-3 binds to TrkC. However, they can also bind to the low affinity neurotrophin receptor p75 (p75NTR) and induce programmed cell death. These opposing effects of neurotrophic factors are important for regulating RGCs development [80,81]. The distribution of neurotrophic factors and their receptors in the mammalian retina has been studied in detail in physiology as well as in pathological conditions [52,53]. Of interest, especially when using in vitro models to study these mechanisms, the expression of neurotrophic factors and their receptors is preserved in glial cells and in RGCs even when in culture for 6 days [86] and the factors secreted by Müller cells offer protection to cultured RGCs [89].

##### Nerve Growth Factor (NGF)

NGF is an important growth factor affecting the survival of nerve cells and their deprivation can lead to apoptosis [90,91]. NGF is produced and utilized by RGCs [92] and protects these cells after injury [93,94,95]. Furthermore, NGF treatments reduced the progressive loss of RGCs in a glaucoma model [93]. In addition, in patients with glaucoma, NGF eye drops resulted in an improvement of the INL function, neural conduction, visual field, optic nerve function, contrast sensitivity, and visual acuity [96]. However, further studies are required to confirm the therapeutic efficacy of NGF.

##### Brain-Derived Neurotrophic Factor (BDNF)

BDNF is widely expressed throughout the CNS. RGCs express BDNF and its high affinity receptor TrkB [97]. As mentioned above, it is a powerful neuroprotective agent that promotes the survival and regrowth of RGCs [61,62,98]. The exposure to NMDA induces an increase in BDNF expression in RGCs in the first hours, suggesting that it is an endogenous neuroprotective response of RGCs. However, this effect is not sustained over time, maybe because the cells cannot maintain the synthesis of BDNF or because the activation of the apoptotic mechanism inhibits BDNF synthesis [52,84]. It has been speculated that the therapeutic properties of different neuroprotective agents in promoting RGC survival are related to the induction of retinal BDNF expression [99,100]. Consistently, BDNF levels are reduced in the serum and tears of glaucoma patients, suggesting that deficits in this neurotrophin may participate in RGC death in glaucoma and that BDNF may be a biomarker for glaucoma [101,102].

##### Glial Cell Line-Derived Neurotrophic Factor (GDNF)

GDNF is secreted by glial cells and binds to the GDNF-α receptor and to the receptor tyrosine kinase in RGCs [103]. GDNF promotes the survival of RGCs after injury [104,105,106]. Moreover, GDNF treatments, specifically intravitreal injection of microspheres containing GDNF, protect RGCs in glaucoma animal models [107,108]. This neuroprotective property of GDNF may be orchestrated by Müller cells. GDNF upregulates the glutamate/aspartate transporter (GLAST) in Müller cells enhancing glutamate uptake that may indirectly protect RGCs [109]. Another possible mechanism of action could be through osteopontin since activation of Müller cells by GDNF was shown to induce the secretion of osteopontin [110]. Thus, GDNF holds strong therapeutic potential for retinal neurodegenerative diseases.

##### Ciliary Neurotrophic Factor (CNTF)

CNTF belongs to the interleukin-6 (IL-6) family of cytokines, binds to CNTF receptors (CNTFR) and exerts robust neuroprotection in neurons [81,111]. In the retina, CNTF is expressed by various cell types, particularly by Müller cells [112]. Its neuroprotective effects are mediated especially by these glial cells that directly respond to CNTF by releasing other neurotrophic factors such as basic fibroblast growth factor (bFGF) [113]. The neurotrophic properties of CNTF were tested in several animal models of glaucoma and in ischemic optic neuropathy [60,114,115,116]. CNTF is also capable of stimulating axonal regeneration [117], which may be mediated by astrocytes [118]. Notably, the concentration of CNTF in the aqueous humor, lacrimal fluid and blood serum is decreased in patients with glaucoma [119]. The results of CNTF in neuroprotection and regeneration suggest a potential for clinical use; however, the pharmacology and administration of CNTF must be optimized.

##### Other Trophic Factors

Other trophic factors have been described to promote RGCs survival. Pigment epithelium derived factor (PEDF) reduces RGC loss in a mouse model of glaucoma [120] and insulin-like growth factor-1 (IGF-1) also protects RGCs from different injuries [121,122]. Despite the role of vascular endothelial growth factor A (VEGF-A) in neovascularization, VEGF is also a neuronal trophic factor that may play a role in RGC neuroprotection. Indeed, it reduces RGC apoptosis in models of glaucoma and anti-VEGF therapies exacerbate neuronal cell death [123,124,125,126].

#### 3.1.2. Glutamate Receptors Antagonists

Despite being the major excitatory neurotransmitter in the retina and involved in the vertical pathway of information [1], excessive glutamate levels have detrimental effects on RGCs [127], a term described as glutamate excitotoxicity [128], due to the activation of a complex apoptotic cascades [128,129]. The fact that intraocular glutamate levels are increased in glaucoma patients [130,131] raised the hypothesis that the blockade of glutamate receptors could be a valuable strategy for RGC neuroprotection, at least for glaucoma. MK801 (dizocilpine maleate) is a potent glutamate receptor antagonist and is a neuroprotective agent of RGCs [132,133], although it could also be neurotoxic [134]. In preclinical studies memantine, a NMDA receptor antagonist, affords robust neuroprotection of RGCs against glutamate toxicity [129,135]. However, memantine had limited efficacy in glaucoma patients [136,137]. More studies are required to clearly evaluate these and other glutamate antagonists as effective neuroprotective therapies for RGCs.

#### 3.1.3. Alpha-2 Adrenergic Receptors Agonists

The presence of alpha-adrenergic receptors in the RGCs has been demonstrated [138]. Additionally, the activation of alpha-2 adrenergic receptors by agonists such as brimonidine has been shown to enhance survival of RGCs after different types of injuries, namely in glaucoma [138,139,140,141], ONC [142] and ischemia [143]. Brimonidine can confer protection by reducing the accumulation of extracellular glutamate and by blocking NMDA receptors, independently of the IOP-lowering mechanisms [139,140,142,144]. Several pre-clinical and clinical studies were conducted [139,145,146,147,148] to assess the protective properties of brimonidine.

#### 3.1.4. Calcium Channel Blockers

Calcium channel blockers may protect RGCs by preventing cell death mediated by calcium influx secondary to NMDA receptor overactivation and local ischemia [149,150]. Different calcium channel blockers attenuate injury to RGCs [151] and increase the viability of immunopurified RGCs cultures [152]. A randomized clinical trial analyzed the effects of the treatment with nilvadipine, a calcium channel blocker, on visual field performance and ocular circulation in patients with open-angle glaucoma. Nilvadipine slowed visual field progression, maintained the optic disc rim, and increased the posterior choroidal circulation [153]. Although these findings look promising, more studies on the distribution and pharmacology of the several types of calcium channels could help clarifying their therapeutic value [154].

#### 3.1.5. Antioxidants

Oxidative stress occurs when concentrations of reactive oxygen species (ROS) rise above physiological range, and it has been indicated as a potential cause of glaucomatous neurodegeneration [155]. Thus, inhibition of ROS may enhance RGC survival [156,157,158]. Coenzyme Q10, cofactor of the electron transport chain that inhibits the generation of ROS, protects retinal neurons from damage [159,160,161]. Moreover, improvement in visual acuity has also been reported in patients with optic neuropathy after treatment with Q10 [162].

Glutathione (GSH) is decreased in glaucoma patients, suggesting a general compromise of the antioxidative defense [163]. The treatment with vitamin E can ameliorate the decrease in the levels of retinal GSH [164,165]. Consequently, vitamin E-deficient diet is associated with an increase of RGC death related to an increase in lipid peroxidation [166]. Moreover, methane increases the activity of several antioxidant enzymes like superoxide dismutase (SOD), catalase (CAT), glutathione peroxidase (GPx), and the expression of anti-apoptotic genes, which culminate in reduced RGC loss [167]. Overexpression of frataxin induces up-regulation of antioxidant enzymes (such as SOD2, CAT, GPx) and increases RGC survival [168]. Other agents, like crocin, increase the levels of GSH and SOD activity, decreasing ROS and promoting RGC survival [169]. Generally, an increase of SOD and alpha-lipoic acid protects RGCs against oxidative stress damage [170,171]. Therefore, evidence demonstrate that antioxidants may be beneficial for neuroprotection of RGCs [148], but further studies are required to investigate their full potential.

#### 3.1.6. Nitric Oxide Synthase Inhibitors

The levels of nitric oxide (NO) are increased in experimental glaucoma, and evidence shows that NO can result in RGCs degeneration [172,173,174,175]. Moreover, increased expression of nitric oxide synthase (NOS) was detected in different models of RGCs injury [175,176,177]. Additionally, in glaucoma patients the astrocytes of ONH become reactive and may produce high amounts of NO causing neurotoxicity to the axons of RGCs [178,179]. This has raised the hypothesis that the inhibition of NOS, in particular inducible NOS (iNOS), could be neuroprotective by delaying RGCs degeneration [180,181]. However, other studies did not identify a relationship between iNOS and RGCs neurodegeneration [182,183]. More studies are necessary to clarify the role of NOS inhibitors in RGCs protection, helping to clarify this “apparent” discrepancy.

#### 3.1.7. Adenosinergic System

Adenosine can exert both neuroprotective and neurodegenerative actions acting through four types of receptors: A_1_, A_2A_, A_2B_ and A_3_. Adenosine acting on adenosine A_1_ receptor (A_1_R) protects cultured retinal neurons from NMDA-induced cell death [184] by blocking calcium channels in RGCs [185], suggesting that agents directed to A_1_R could be a good therapeutic strategy. Indeed, the activation of A_1_R is neuroprotective against injury induced by ischemia-reperfusion [186], and N(6)-cyclohexyl-adenosine (CHA), an agonist of A_1_R, increases RGCs survival mediating the trophic effect of IL-6 [187]. In fact, IL-6 is an interesting cytokine that has been demonstrated to promote RGCs survival [188,189], probably by the modulation of BDNF synthesis [190]. Adenosine A_3_ receptor (A_3_R) has also been evaluated as a therapeutic target [191]. RGCs are endowed with A_3_R [192], and its activation protects RGCs from cell death induced by P2X7 receptor agonist [193,194], possibly by limiting the rise in intracellular calcium [195]. Activation of A_3_R promotes RGCs neurite outgrowth and neurite regeneration in an animal model of ONC [196]. Moreover, the activation of A_3_R was also demonstrated to afford protection to the retina from excitotoxic-induced cell death, retinal ischemia-reperfusion injury and damage induced by partial optic nerve transection [197].

### 3.2. Cell-Based Therapies

Beyond neuroprotection, cell replacement may have potential as a strategy for the treatment of optic neuropathies. Replacing the diseased or degenerated cells by stem cell-derived RGCs should provide effective therapeutic treatment in the near future. However, complex circuitry in the retina makes cell replacement challenging and difficult for functional repair [198].

Stem cells are functionally undifferentiated and immature cells of a complex nature. These cells are capable of differentiating into different cell types, indicating that they have the potential to repair tissue and restore function after lesion. Due to this potential, it is believed that stem cells may be able to either replace or repair damaged cells in the retina [199,200,201].

In the past decade, the capacity to generate retinal cells from pluripotent stem cells using three-dimensional organoid cultures has become well established [202,203,204]. However, while corneal transplantation is commonly performed with excellent results, many obstacles must be surpassed before retinal transplants can become clinically useful. The major problems are the production of appropriate transplants, functional integration in situ and the survival of the stem cell-derived RGCs.

Various types of stem cells were assessed for retinal differentiation and transplantation such as human embryonic stem cells, induced pluripotent stem cells, isolated retinal stem cells and also from adult stem cells, in particular neural stem cells, mesenchymal stem cells (MSCs) derived from bone marrow, adipose tissues and dental pulp [205].

Various methods to assess the ability of RGCs to survive and integrate with host tissue have been proposed [206,207]. Transplanted RGCs by intravitreal injection acquired the normal morphology of endogenous RGCs, responded to light, and established synaptic contacts with the lateral geniculate nucleus and the superior colliculus [208,209,210]. These examples show that RGC transplantation is possible, although not very efficient, but further studies will certainly guarantee that transplantation of cells to the retina may become a strategy.

MSCs have been widely demonstrated to afford neuroprotective, immunomodulatory and antioxidant properties, making them a promising strategy for the treatment of neurodegenerative diseases. These cells secrete neurotrophic factors like NGF accelerating the survival of neural cells [211]. The protective properties of MSCs have also been also documented in an animal model of glaucoma and in an animal model of optic nerve injury [212,213]. The protective properties of MSCs extend beyond the cells. Recently, the extracellular vesicles derived by these cells were demonstrated to promote RGCs neuroprotection in rodent models of glaucoma [213,214].

### 3.3. Glia-Mediated Neuroprotection

The term neuroinflammation comprises a number of events that affects the CNS. In other words, every time the CNS is faced with infectious agents, traumatic injuries or other unknown elements that might cause a disruption of its homeostasis, it will protect itself by the initiation of inflammatory signaling cascades in order to eliminate the pathological factor [215]. Although the main actors in this scenario are astrocytes and microglia [216,217], in the retina, Müller cells can also be activated and get involved in the production of inflammatory cytokines and chemokines, which maintain and enhance the inflammatory condition participating in the progression of several diseases.

Microglial cells have long been recognized as crucial players in the maintenance of retinal homeostasis. During development, microglial cells are involved in synaptic pruning and in retinal wiring [218] and throughout the life of the organism these cells screen the parenchyma searching for alterations in the environment, including cell interactions and external threats [219,220]. In pathological conditions, microglia have been shown to interfere with neural and glial cell function contributing to retinal degeneration and RGC loss [221]. Indeed, several reports show that abnormally responsive microglia can directly reduce the survival of RGCs. For example, even though microglial cells are not endowed with NMDA receptors, upon intravitreal NMDA injection, these cells detect the alterations in calcium and adenosine triphosphate (ATP) signaling in other retinal cells, including RGCs, by increasing the inflammatory response [222]. Interestingly, the report that isolated RGCs are resistant to NMDA excitotoxicity [223], while in the retina NMDA exposure leads to RGCs degeneration triggered by increased production of tumor necrosis factor (TNF) and abnormal behavior of microglial cells [222] is another evidence of the role of microglial cells shaping RGCs degeneration.

The pivotal role of microglia in RGCs degeneration has been mostly explored in glaucoma. Historically, reactive microglial cells have been associated with human glaucomatous ONH lesion, mainly by their spatial distribution along the damaged fibers and expression of activation markers as well as pro-inflammatory mediators [224,225]. Indeed, enlarged reactive microglial cells were found in the retina of human post-mortem donors with glaucoma manifestations [224]. Nevertheless, this finding may raise the question of whether microgliosis might be a cause or a consequence of the retinal degeneration. This question was very elegantly addressed using the DBA/2J mouse model of glaucoma when microglia activation in the ONH was visualized before the detection of RGC loss [226,227]. Furthermore, microglial cell response initiates in the unmyelinated region of the ONH and further develops along the retina, correlating with the progression of the neurodegenerative process [227]. In accordance with these findings, microglia reactivity was shown to impact RGCs survival in different experimental models of glaucoma [78,79,228]. Altered ROS signalling has been associated with glaucomatous damage both in animal models and in human glaucoma [179,229], and reactive microglial cells may be the main cellular source [79]. In a model of induced ocular hypertension (OHT), microglia were shown to be reactive as detected by the increased expression of translocator protein (TSPO), major histocompatibility complex class II (MHC-II) and pro-inflammatory mediators in the retina early after OHT induction [230]. Even when exposed to elevated hydrostatic pressure (EHP) an in vitro model of elevated IOP, microglia become reactive, release pro-inflammatory mediators and increase ATP and adenosine secretion [78,231]. Alterations in ATP levels are determinant to propagate microglial cell response by acting as a “call for action” [232]. In addition, adenosine mainly acting through the activation of A_2A_ receptor (A_2A_R) may propel microglia deleterious response overtime [233]. The A_2A_R has been described to control microglia reactivity. Its expression increases in microglia in models of glaucoma [78,79], and A_2A_R antagonists were shown to confer protection to retinal neurons, including RGCs, through the control of microglia reactivity [78,79,228]. Caffeine, a non-selective adenosine antagonist, also protects RGCs by hampering microglial cell response and controlling the neuroinflammatory environment in models of transient retinal ischemia and ocular hypertension [230,234,235]. KW6002, another A_2A_R antagonist with good oral bioavailability, confers protection to the retina, including RGCs, through the control of microglia-mediated neuroinflammation [234]. Recently, the potential of A_2A_R antagonists was further confirmed as a strategy for the human retina [78]. By using human retinal organotypic cultures, the A_2A_R antagonist was able to reduce microglia alterations and the production of ROS, suggesting that microglia-mediated inflammation in the human retina also involves A_2A_R [78]. The neutralization of the actions of TNF and interleukin-1β (IL-1β) in the retinal organotypic cultures was able to prevent the loss of RGCs triggered by EHP, reinforcing the role of retinal inflammation in neurodegeneration in glaucoma [79]. The central role of microglia causing RGCs loss was further demonstrated with a strategy to deplete microglia from primary retinal cultures following exposure to EHP [78]. In such case, the effect of EHP on cell death was abrogated, showing that microglia are indeed the main triggers and propellants of neuroinflammation-mediated glaucomatous damage [78].

The secretion of TNF by microglial cells was shown to contribute to RGC degeneration as its receptor is highly increased in glaucoma in RGCs, astrocytes, microglia and Müller cells, triggering a cascade of events that culminates in RGC demise [236,237]. Indeed, simple experiments neutralizing the actions of TNF were able to restore axon function and decrease the loss of RGCs in glaucoma [238].

Recently it has been shown that chronic OHT promotes the expression of P2X7 receptor in the retina leading to the activation of NLRP3 (NOD-, LRR- and pyrin domain-containing protein 3) inflammasome [239]. The activation of P2X7R-dependent NLRP3 inflammasome in microglia increases the production of pro-inflammatory cytokines and caspase activation that leads to RGC death [239]. In accordance with the role of purine receptors in microglia reactivity, the inhibition of P2X7 receptor in microglial cells confers protection to RGCs in vitro upon exposure to conditioned media from microglia exposed to BzATP by decreasing NLRP3 inflammasome activation [239]. Furthermore, in a model of OHT the oral administration of a saffron extract reduced retinal microglia inflammation and the loss of RGCs, while decreasing the expression of the adenosine diphosphate P2Y12 receptor [240].

Microglial cells also modulate retinal cell function by expressing complement molecules. In fact, in human and experimental glaucoma the expression of complement factors is increased in conditions of elevated IOP [241,242,243]. The complement proteins C1q and C3 are crucial during retinal development by allowing the targeting of dysfunctional or unnecessary synapses to prune by microglial cells [244,245]. However, in glaucoma the inadequate targeting of synapses by increased expression of complement factors by microglia leads to indiscriminate pruning of healthy neurons, which might contribute to disease progression [246]. In addition, in glaucoma, microglia were found to actively phagocyte functional RGCs increasing the loss of visual capacity [246]. Furthermore, if neuron-microglia communication is impaired by interfering with the fractalkine receptor (CX3C chemokine receptor 1, Cx3cr1) in microglia, this would aggravate RGC loss in disease models such the ischemia-reperfusion [247] and glaucoma DBA/2J mouse [248], with no alterations in uninjured retinas [247]. These findings suggest that in the context of disease although the control of microglia response might be beneficial, it is important to preserve cell communication to restrain microglial cell response. Therefore, a strategy to confer protection to retinal cells might be to block the over targeting of retinal neurons by microglia through the complement system [241].

In the model of ischemia-reperfusion, treatment with minocycline, which decreases microglia reactivity, was able to protect RGCs [247]. Moreover, in the DBA/2J mouse model of glaucoma, minocycline decreases the number of ameboid microglia increasing their ramification and reducing the neuroinflammatory milieu [249]. Moreover, minocycline also improved axonal transport in RGCs and overall retinal integrity in the glaucoma model [249], providing evidence that the control of microglia-mediated neuroinflammation can have potential in RGC neuroprotection.

Müller cells are the main glial cells in the retina. In addition to structural support, among other functions, Müller cells are involved in metabolism, phagocytosis of neuronal debris and in the release of trophic factors. These cells can enhance the survival of RGCs [89,250,251,252].

**Figure 5:**
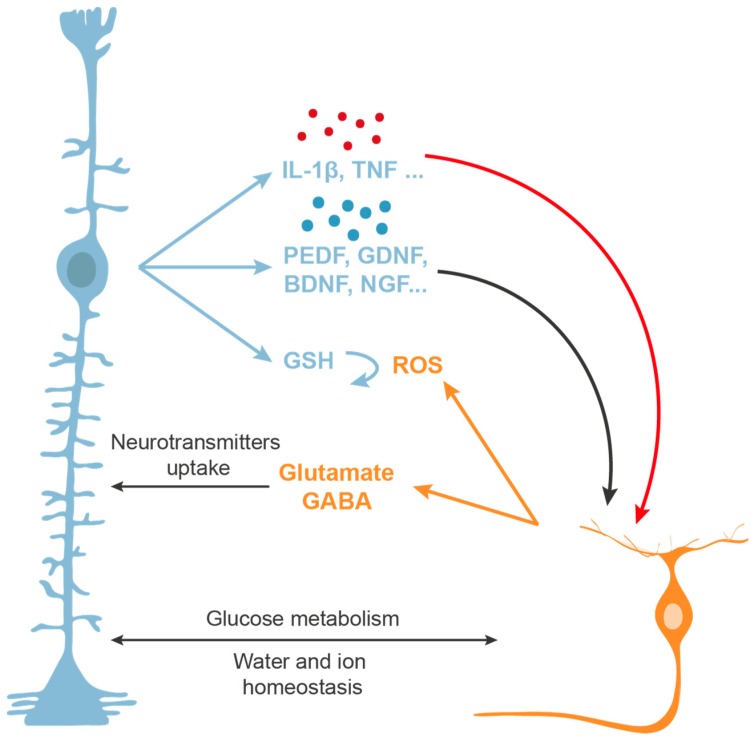
Diagram summarizing the main interactions of Müller cells (blue) with RGCs (orange). Scheme showing the roles of Müller cells in RGC neuroprotection, such as glucose metabolism regulation, water and ion homeostasis, neurotransmitters uptake, antioxidant defense systems (GSH) against ROS, secretion of trophic factors. The role of Müller cells in inflammation by secretion of cytokines that may be detrimental for RGCs is also depicted (red arrow).

Müller cells are crucial to protect neurons against toxic molecules (Figure 5). They can uptake excessive glutamate from the synapses, preventing glutamate-induced RGC death [253,254]. Some studies have demonstrated this function of Müller cells in vivo as well as in vitro [255,256,257,258]. The glutamate transporter GLAST contributes to the uptake of excess of glutamate from the medium protecting against the excitotoxic effect of glutamate [254,256]. Moreover, it has been shown that in some ocular diseases, the expression of GLAST is altered, including in an animal model of glaucoma [259]. Moreover, Müller cells are implicated in maintaining the retinal extracellular levels of other neurotransmitters, such as gamma-aminobutyric acid (GABA), contributing to neuronal protection [260].

Müller cells are also involved in the regulation of glycogen and glucose metabolism and during metabolic stress can provide lactate to retinal neurons [261]. For instance, in early phases of diabetic retinopathy, Müller glia may afford neuroprotection against high glucose [262]. In addition, due to their energy metabolism, Müller cells may protect neurons towards toxic stress by increasing ATP turnover [263]. Furthermore, they play a role in water and ion regulation, buffering the retina and inducing neuroprotection [250,264]. It is worth highlighting the important antioxidant role of Müller cells. One crucial molecule that protects the retina against reactive oxygen species is GSH, which can be synthesized by Müller cells [254,265]. In addition, GSH can prevent RGC degeneration in an experimental model of glaucoma [266].

Müller cells secrete a great number of factors in response to injury that can protect retinal neurons. Müller cells are known to synthesize neurotrophins and growth factors that can increase RGC survival [53,86,252,254], such as PEDF [267] or GDNF [105] among others. Moreover, Müller cells produce selective neurotrophins under different conditions, for instance, in response to glutamate, these cells upregulate the secretion of BDNF, NGF, NT-3, NT-4, and GDNF [268]. Müller cells are not only a source of neurotrophins, they also respond to neurotrophins as they express neurotrophin receptors [53,269] (see the neurotrophic factors functions protecting RGCs above).

Furthermore, Müller glial cells can release several inflammatory factors and cytokines [270], and some cytokines are even known to stimulate the production of other cytokines by Müller glia [271] in response to different stressors. Müller cells are a major source of retinal IL-1β [272,273] and they also secrete TNF, facilitating the apoptotic death of RGCs in response to damage [274,275]. In addition, Müller cells express toll-like receptors (TLRs) [276] and receptors for advanced glycation end-products (RAGE) [277] that induce the production of pro-inflammatory cytokines, chemokines and neuroprotective growth factors by these cells. In the beginning, this process acts as a protective mechanism to prevent further damage to the retina and to promote tissue repair. However, in the adult mammalian retina it does not appear to be beneficial since the release of pro-inflammatory cytokines and growth factors from Müller cells can lead to further degeneration [278]. For these reasons, understanding the processes in which Müller cells are involved and how these processes differ between pathological conditions and finding strategies to circumvent these barriers represent major challenges to the advancement of many ocular therapies.

## 4. Clinical Trials Targeting RGCs Neuroprotection

A therapeutic strategy to optic neuropathies should protect RGCs from death but should also manipulate axonal regeneration in order to repair the visual function that was lost due to the disease. However, there is still no effective therapy for optic neuropathies. Innovative study designs and integrating therapeutic testing with biomarkers have advanced several neuroprotective and neuroenhancement compounds to clinical trials. Numerous neuroprotection strategies have been investigated for optic neuropathies, including peripheral nerve grafting, electrical stimulation, and in agreement with their well-known role in maintaining neuronal homeostasis, neurotrophic factors have been proposed as a novel therapy. However, the outcomes of the completed clinical trials were not completely satisfactory, presenting only partial or no expected effects [198,279,280,281].

There are several drugs in clinical trials that are currently being developed focused on RGC neuroprotection (Table 1). In the context of neurotrophic factors some clinical trials are available. NT-501 encapsulated cell therapy (NT-501 ECT) is a device produced by Neurotech that consists of an intravitreal implant with a capsule filled with human cells genetically modified to secrete CNTF. NT-501 ECT is in phase 2 for glaucoma (ClinicalTrials.gov Identifier: NCT02862938) and in phase 1 for ischemic optic neuropathy (ClinicalTrials.gov Identifier: NCT01411657). For glaucoma, other therapies have been proposed such as the use of recombinant human NGF (rhNGF) (ClinicalTrials.gov Identifier: NCT02855450). In this phase 1 clinical trial the safety and tolerability of an 8-week treatment with 180 μg/mL of rhNGF eye drop solution will be determined. Additionally, the study wants to assess the changes in best corrected distance visual acuity (BCDVA), visual field, electroretinography (ERG) and structural changes in GCL and NFL thickness measured by optical coherence tomography (OCT) at 1, 4 and 8 weeks of therapy, and at 4 and 24 weeks after therapy cessation. In another clinical trial the safety of treatment with single and multiple ascending doses of rhNGF (0.5–180 µg/mL) was tested in healthy patients (ClinicalTrials.gov Identifier: NCT01744704), and the results demonstrated that rhNGF eye drops were well tolerated by the patients [282].

The only modifiable risk factor for glaucoma development is elevated IOP. Brimonidine is a non-selective α2-adrenergic receptor agonist and is currently used as a treatment option in glaucoma to lower IOP [283]. Preclinical studies demonstrated the neuroprotective properties of brimonidine [143,284], leading to the hypothesis that an implant with brimonidine can have beneficial properties for glaucoma patients. Indeed, this device is being evaluated in patients with glaucomatous optic neuropathy (ClinicalTrials.gov Identifier: NCT00693485). Moreover, cytidine-5′-diphosphocholine (citicoline) is also in a phase 4 clinical trial for glaucoma (ClinicalTrials.gov Identifier: NCT00404729). Citicoline is an endogenous molecule that has a role in the biosynthesis of phospholipids of cell membranes and increases the levels of neurotransmitters, like acetylcholine, in the CNS [285]. The neuroprotective properties of citicoline in glaucoma have been tested [286,287]. Intramuscular treatment of citicoline improves glaucomatous visual defects [286], RGC function (assessed by pattern ERG) and neural conduction along postretinal visual pathways (assessed by visual-evoked potential) [288]. That way, the phase 4 clinical trial aims to assess the effects of oral citicoline treatment in visual function outcomes in glaucoma patients. Memantine, a NMDA subtype of glutamate receptor antagonist, is already being used for Alzheimer’s disease, and has undergone phase 3 clinical trials for glaucoma (ClinicalTrials.gov Identifier: NCT00141882 and NCT00168350). However, the drug did not show significant efficacy in preserving visual function in glaucoma patients [289].

Moreover, prostaglandin E1 (alprostadil) administered by intravenous infusion, is very recently in phase 2 clinical trial (ClinicalTrials.gov Identifier: NCT03851562). Prostaglandin E1 is a potent vasodilator of the microcirculation [290], and may correct the deficits in the perfusion pressure of the microcirculation that supplies the optic nerve in patients with ischemic optic neuropathy, improving visual function. In fact, intravenous prostaglandin E1 is an effective treatment for ocular and optic nerve ischemia leading to immediate visual improvement [290]. On the other hand, due to the role of endothelin in glaucoma as a potent vasoconstrictor [291], the antagonism of its signaling seems to be a good therapeutic strategy for optic neuropathies. Bosentan, an endothelin receptor antagonist, is in phase 3 clinical trial for ischemic optic neuropathy in order to assess if the treatment could recover anatomical (NFL in OCT, optic atrophy) and functional (visual acuity, visual field) criteria (ClinicalTrials.gov Identifier: NCT02377271). The last drug-based therapy for ischemic optic neuropathy, the retrobulbar injection of triamcinolone acetonide to halt the progression of the visual acuity and visual field loss in patients improving their chances of avoiding blindness, is in phase 3 clinical trial (ClinicalTrials.gov Identifier: NCT02329288). In preclinical studies, besides the neuroprotective effects to RGCs conferred by triamcinolone acetonide, it was demonstrated that this drug also decreases the activation of retinal microglia [292]. For non-arteritic ischemic optic neuropathy there are several clinical trials targeting neuroprotection. EPO administered by intravenous injection started recently in phase 2 clinical trial, in order to assess visual field and thickness of the retinal NFL by OCT in glaucoma patients (ClinicalTrials.gov Identifier: NCT03715881). In the same clinical trial, another aim is to assess the potential retinal neuroprotective effect of prednisolone. Moreover, methylprednisolone is also in phase 3 clinical trial (ClinicalTrials.gov Identifier: NCT02439866). Preclinical studies demonstrated that methylprednisolone inhibits the apoptosis of RGCs after ONC, probably through an up-regulation of Bcl-2 expression and a down-regulation of Bax expression [293], two of the intrinsic factors that limit the axon regeneration described previously. Moreover, citicoline is in clinical trials for non-arteritic ischemic optic neuropathy (ClinicalTrials.gov Identifier: NCT03046693) in order to assess the function of RGCs by pattern ERG, thickness of GCL and visual field test.

RPh201 is a drug extracted from a botanical source and it has been produced by Regenera Pharma. RPh201 started recently the phase 3 clinical trial for non-arteritic ischemic optic neuropathy (ClinicalTrials.gov Identifier: NCT03547206). The results of the phase 2 clinical trial (ClinicalTrials.gov Identifier: NCT02045212) are already available. Patients showed an improvement in visual function after the treatment [294]. Dalfampridine is used to improve the walking ability in multiple sclerosis patients and is in a phase 4 clinical trial for non-arteritic ischemic optic neuropathy (ClinicalTrials.gov Identifier: NCT01975324).

Anti-VEGF antibodies (bevacizumab, avastin or ranibizumab) are used for the treatment of macular edema and neovascular age-related macular degeneration. However, they have also been tested for neuroprotection in optic neuropathies, and they are in three different clinical trials for non-arteritic anterior ischemic optic neuropathy (ClinicalTrials.gov Identifier: NCT01330524, NCT00813059 and NCT00561834) in order to halt the progression of visual acuity and visual field loss due to the disease. The thickness of GCL increased after the treatment with bevacizumab in diabetic macular edema [295]. Moreover, levodopa-carbidopa is used to treat the symptoms of Parkinson’s disease and it is in a phase 4 clinical trial for non-arteritic anterior ischemic optic neuropathy (ClinicalTrials.gov Identifier: NCT00432393).

A phase 1 and 2 clinical trial (ClinicalTrials.gov Identifier: NCT01783847) assessing the effect of erythropoietin (EPO) demonstrated an improvement in visual function [296,297]. These beneficial effects can be due to the protection conferred to RGCs by EPO previously demonstrated in animal models of retinal degeneration [298]. Moreover, it has been tested whether EPO could improve optic nerve function and help patients to recover visual function after methanol associated optic neuropathy (ClinicalTrials.gov Identifier: NCT02376881). EPO is currently in phase 3 clinical trial for traumatic optic neuropathy (ClinicalTrials.gov Identifier: NCT03308448).

Leber’s hereditary optic neuropathy is an inherited optic neuropathy characterized by mitochondrial dysfunction that leads to vision loss due to RGCs loss [299]. Idebenone was in clinical trials for the treatment of vision loss due to Leber’s hereditary optic neuropathy (ClinicalTrials.gov Identifier: NCT02774005 and NCT00747487). The beneficial effects of idebenone are due to its antioxidant properties and its ability to act as an electron carrier in the mitochondrial respiratory chain, thus resulting in the restoration of cellular energy (ATP) generation and contributing to the recovery of visual function in patients (reviewed in [300]). That way, idebenone (Raxone^®^) is the first, and currently the only disease-specific treatment for Leber’s hereditary optic neuropathy and the only approved for optic neuropathies aiming RGCs neuroprotection. Moreover, cyclosporine is also in a phase 2 clinical trial for Leber’s hereditary optic neuropathy (ClinicalTrials.gov Identifier: NCT02176733), due to protective properties against ischemic injury-mediated mitochondrial dysfunction in RGCs [301].

Currently, there are two clinical trials involving stem-cell based therapies targeting RGCs (Table 2). One trial aims to assess the safety and efficacy of the transplantation of autologous purified stem cells (ClinicalTrials.gov Identifier: NCT02638714) on restoring function in damaged optic nerves using autologous purified populations of bone-marrow derived stem cells in optic neuropathy. The intravitreal injection of MSCs (ClinicalTrials.gov Identifier: NCT03173638) aims to evaluate if the treatment may reduce the progression of axonal degeneration caused by non-arteritic ischemic optic neuropathy, but this clinical trial is focused in the evaluation of the safety of cell therapy as a new treatment for these patients.

## 5. Different Types of RGCs and their Susceptibility after Retinal Damage

The complexity of the CNS is due to the great number of specialized neuronal types and subtypes that give rise to a complex connectome [302]. However, due to the heterogeneity and complexity of the mammalian neuronal types, neuronal classification has been challenging and many cell subtypes have not yet been characterized [303]. Just like neurons in the brain, in the retina, although most RGCs serve a similar function, it was proven that these RGCs are highly diverse. The total RGC population develops from a common precursor into different subtypes of RGCs, with that they may differ in their physiological roles generating varied responses to visual stimuli [304]. RGCs have been classified based on differences in size, morphology, dendritic arborization, electrophysiological functions, susceptibility to degeneration, regenerative capacity and expression of specific molecular signatures, and more than thirty different subtypes of RGCs subtypes have been identified to date in the mammalian retina [305]. In 1953, the first classifications were made and the ON- and OFF- center RGCs were distinguished [305]. In recent works, combining different criteria, RGCs were classified into four types of ON-OFF directionally selective ganglion cells (DS-RGCs), three types of ON DS-RGCs, three types of alpha RGCs (sustained ON, sustained OFF, and transient OFF αRGCs), five types of intrinsically photosensitive melanopsin-containing RGCs (ipRGCs), three types of JamB expressing RGCs (J-RGCs), two types of beta cells (βRGCs), chromatically sensitive ganglion cells, orientation-sensitive cells, and suppressed-by-contrast cells, among others [306].

The major requirement to properly characterize and classify RGCs is to distinguish selectively each specific subtype. Thus, recently, a number of molecular subtype-specific markers have been described to further classify different subtypes of RGCs [307]. Several markers are being proposed, but most of them sign more than a single RGC subtype. Four types of ON–OFF DS-RGCs have been described, depending on the direction of the moving object to which they respond. They all have similar dendritic stratification and express CART (cocaine and amphetamine-regulated transcript) [308]. DS-RGCs have been also identified by the expression of specific molecular markers, such as CDH6 and FSTL4 [307]. Moreover, the ON DS-RGCs can be identified by the expression of the secretory protein SPIG1 [309]. In addition, three types of α-RGCs express similar markers, including neurofilaments, spp1 and kcng4, among others [310], although they differ in their physiological properties, dendritic arborization and stratification in the IPL [306]. On the other hand, the ipRGCs mediate many relevant non-image forming functions of the eye and they are identified by the expression of the photopigment melanopsin [311].

Another molecular marker, Tbr2, identifies RGCs that are hardwired during developmental stages and they could be precursors of ipRGCs [312]. Other single subtype of RGCs appears to be uniquely marked by the transcription factor Prdm16. However, the precise identity of these RGCs is unclear, but they most resemble the G9 subtype described by Völgyi and colleagues in 2009 [313].

Moreover, mouse transgenic lines have been also used to label and identify specific subsets of RGCs [314]. For instance, the line CB2-GFP labels transient OFF αRGCs [315] and the line Isl2-GFP labels αRGCs but not ON-OFF DS-RGCs [316]. In addition, using single cell transcriptome profiling of RGCs, specific markers for cellular subtypes have been identified, such as Zic1, Runx1 and Fst [317]. This capacity to successfully identify RGCs subtypes hopefully will help to understand the different susceptibility of certain RGCs to progression of pathologies like glaucoma.

In order to understand the pathophysiology of neurodegenerative diseases in which the RGCs death is implicated, it is important to analyze the response of these RGCs subtypes individually rather than studying them as a single entity. RGCs are susceptible to various injuries in a type-specific manner. Thereby, their type-specific vulnerability has been extensively studied [318]. The identification and characterization of the loss of specific RGCs subtypes in axotomy [310], ONC [319] and glaucoma [320] models has been analyzed, suggesting subtype specific responses to injury. The importance of studying the response of these subtypes individually rather than studying them as a single entity could help us understand the pathophysiology of diseases in which RGCs are affected. For instance, it was found that a greater loss of large RGCs in the peripheral retina occur in a pig glaucoma model resembling what was described in glaucoma patients [321]. However, in the periphery of the retina, some cells are resistant to damage, and it will be very important to know the nature of the cells that possess the capacity to recover after an insult. The αRGCs seem to be the least susceptible RGCs subtype to optic nerve injury [310]. The αRGCs are also the most resistant RGCs to NMDA excitotoxicity, while the J-RGCs are the most sensitive to the same damage [322]. Nevertheless, αRGCs seem to be the more susceptible RGCs subtype in other studies, such as in autoimmune optic neuritis, where αRGCs are more vulnerable to degeneration than ON αRGCs [323], and after ONC injury, where OFF-transient αRGCs are the most susceptible to injury followed by ON-OFF DS-RGCs [319]. In experimental models of OHT, OFF-transient RGCs exhibited a faster decline on survival when compared to ON RGCs, and they were also the first to undergo structural alterations [318]. Similarly, after ONC injury, functional responses and receptive fields of OFF cells were also impaired earlier than ON cells, and ON sustained RGCs seem to be more susceptible than ON transient RGCs [324]. In another model of OHT, the mono-laminated ON RGCs were more susceptible to chronic OHT than bi-laminated ON-OFF cells [325].

It has been shown that non-image forming ipRGCs exhibited a preferential survival following injury compared to image forming RGCs. This fact was observed in different injury and disease models, demonstrating the resilience to damage of this subtype of RGCs [326]. ipRGCs have the ability to respond to light using the photopigment melanopsin, and they play a role in circadian rhythms and pupillary reflexes through their projections to the suprachiasmatic nucleus and the olivary pretectal nucleus [327]. This unique feature may be the basis of their resistance to insult, as these cells are not necessary for the formation of images in the visual transduction pathway [328].

All these studies clearly imply that RGCs respond in a subtype specific manner to injury. Moreover, each subtype of RGCs can have a unique gene expression pattern [306,329], this differential gene expression may protect some types of RGCs and facilitate the death of others [330]. Therefore, the analysis of the type-specific vulnerability of RGCs based on their gene expression may provide insights of the selective vulnerability of RGCs to pathological insults and to better understand disease mechanisms [322]. Moreover, further studies are needed to determine how the molecular differences between RGCs subtypes underlie their electrophysiological functions, and it is necessary to investigate whether they differ in morphology, retinal spatial distribution, target cell connectivity, and associated visual parameters. These studies could provide a new opportunity to the development of strategies to target specific subtypes of RGCs for diagnostic and therapeutic approaches to treat optic neuropathies, such as glaucoma.

## 6. Potential Pitfalls in Translating Preclinical Studies into the Clinics

The main goal in finding new therapeutic strategies for optic neuropathies is to preserve the function of RGCs in order to maintain visual pathways. Therefore, besides the neuroprotection of RGCs and axon regeneration, the re-integration of RGC axons into the appropriate visual circuity is also important. Despite that several therapeutic strategies have demonstrated promising results in this field, there are significant issues affecting their translation to clinical practice.

Much work has been done in order to identify the inhibitors of axonal growth in the CNS as well as to isolate neurotrophic factors, with the hope that one day these factors could be applied to protect and regenerate the optic nerve. From what was described above, it seems that we are getting closer to a therapeutic strategy focused on RGC neuroprotection for optic neuropathies. However, we can discuss the example of memantine that, despite the convincing neuroprotective effects in animal models of glaucoma, in clinical trials the drug did not reveal significant effects in preventing the progression of visual field loss in patients with glaucoma [289].

Several issues contributing to the lack of success of drugs in clinical trials could be suggested, but in the case of glaucoma, the lack of an animal model that fully mimics the human disease is an important factor that adds to this failure [331]. Another issue is that, in preclinical trials, several studies use a preventive strategy to assess the effect of a specific drug, as opposed to the human condition in which the treatment starts after diagnosis. Moreover, in most of the animal studies the evaluation of the drug beneficial effects occurs by histopathological methodologies, and this is not possible in human studies. The increasingly use of OCT and ERG in preclinical studies will benefit the translation of what is observed in an animal models of disease into human.

Besides the protection of RGCs from death and degeneration, one of the goals in RGCs regeneration therapies should be to allow the reintegration of the regenerating axons into visual circuity reaching the appropriate brain targets. However, there are few studies that focus on this issue [26]. Moreover, the identification of different types of RGCs and the characterization of their different susceptibility to disease [306,324] may also contribute to the failure of the therapeutic strategies with high potential of success in the clinical trials phase.

It is fundamental a better characterization of the beneficial effects of drugs in the preclinical phase, meaning that the observation of the loss of RGCs is not enough as it is not enough to observe potential regenerative events of the axons of RGCs. It is essential to clearly and more deeply evaluate the beneficial effects of a specific new drug also in visual function.

The research in the field of neuroprotection in glaucoma has been difficult, but new animal models of disease and techniques will help to bridge the gap between preclinical and clinical studies, with clear beneficial outcomes in the forthcoming years. Many of the approaches outlined in this review are applicable not only to RGC neuroprotection in glaucoma but also to other pathologies of the optic nerve and retina. Gene therapy may have also a therapeutic potential especially for Leber’s hereditary optic neuropathy, an optic neuropathy caused by mitochondrial mutation G11778A in NADH dehydrogenase subunit 4 (ND4) gene [332]. It was conducted in patients and the recombinant adeno-associated virus 2 (AAV2) carrying ND4 (rAAV2-ND4) demonstrated to improve patients visual acuity [333,334,335]. In addition, CRISPR/Cas9-based therapies are starting to be applied making significant progress in mammalian preclinical models of eye disease such as blind rodents [336] or in the disruption of mutant genes that cause certain forms of glaucoma [337]. Applications of CRISPR/Cas9 technology and other gene therapies may soon be available, not only as research tools but also as therapies to treat retinal diseases.

## Figures and Tables

**Table 1:** Drug-based therapies in clinical trials for optic neuropathies.

Condition or Disease	Intervention	ClinicalTrials.gov Identifier	Phase	Starting Date
Glaucoma	NT-501 ECT implant	NCT02862938	2	2016
Glaucoma	rhNGF	NCT02855450	1	2016
Glaucoma, Primary Open Angle	NT-501 CNTF Implant	NCT01408472	1	2011
Glaucoma, Open-Angle	Brimonidine Implant	NCT00693485	2	2008
Glaucoma and Ischemic optic neuropathy	Citicoline	NCT00404729	4	2006
Open-Angle Glaucoma	Memantine	NCT00141882	3	2005
Open-Angle Glaucoma	Memantine	NCT00168350	3	2005
Ischemic Optic Neuropathy	Alprostadil (prostaglandin E1)	NCT03851562	2	2019
Ischemic Optic Neuropathy	Bosentan	NCT02377271	3	2015
Ischemic Optic Neuropathy	Triamcinolone Acetonide	NCT02329288	3	2014
Ischemic Optic Neuropathy	NT-501 CNTF Implant	NCT01411657	1	2011
Non-arteritic Anterior Ischemic Optic Neuropathy	Prednisolone and Erythropoietin	NCT03715881	2	2018
Non-arteritic Ischemic Optic Neuropathy	RPh201	NCT03547206	3	2018
Non-arteritic Anterior Ischemic Optic Neuropathy	Citicoline	NCT03046693	4	2017
Non-arteritic Anterior Ischemic Optic Neuropathy	Methylprednisolone	NCT02439866	3	2015
Non-arteritic Ischemic Optic Neuropathy	RPh201	NCT02045212	2	2014
Non-arteritic Ischemic Optic Neuropathy	Dalfampridine	NCT01975324	4	2013
Non-arteritic Anterior Ischemic Optic Neuropathy	Avastin and Triamcinolone	NCT01330524	1 and 2	2011
Non-arteritic Anterior Ischemic Optic Neuropathy	Bevacizumab	NCT00813059	2	2008
Non-arteritic Anterior Ischemic Optic Neuropathy	Ranibizumab	NCT00561834	1	2007
Non-arteritic Anterior Ischemic Optic Neuropathy	Levodopa-carbidopa	NCT00432393	4	2007
Traumatic Optic Neuropathy	Recombinant human erythropoietin	NCT03308448	3	2017
Traumatic Optic Neuropathy	Recombinant human erythropoietin	NCT01783847	1 and 2	2013
Optic Nerve Diseases (methanol associated optic neuropathy)	Erythropoietin	NCT02376881	3	2015
Leber’s Hereditary Optic Neuropathy	Idebenone	NCT02774005	4	2016
Leber’s Hereditary Optic Neuropathy	Cyclosporine	NCT02176733	2	2014
Leber’s Hereditary Optic Neuropathy	Idebenone	NCT00747487	2	2008

**Table 2:** Stem cell-based therapies in clinical trials for optic neuropathies.

Condition or Disease	Intervention	ClinicalTrials.gov Identifier	Phase	Starting Date
Optic Neuropathy	Transplantation of autologous purified stem cells	NCT02638714	1 and 2	2015
Non-arteritic Ischemic Optic Neuropathy	Intravitreal injection of mesenchymal stem cells	NCT03173638	2	2017

## Abbreviations

A_1_	Adenosine A_1_ receptor
A_2A_	Adenosine A_2A_ receptor
A_2B_	Adenosine A_2B_ receptor
A_3_	Adenosine A_3_ receptor
ARTN	Artemin
ATP	Adenosine triphosphate
BCDVA	Best corrected distance visual acuity
BDNF	Brain-derived neurotrophic factor
bFGF	Basic fibroblast growth factor
cAMP	Cyclic adenosine monophosphate
CART	Cocaine and amphetamine-regulated transcript
CAT	Catalase
CHA	N(6)-cyclohexyl-adenosine
CNS	Central nervous system
CNTF	Ciliary neurotrophic factor
CNTFR	CNTF receptors
Cop-1	Copolymer-1
Cx3cr1	CX3C chemokine receptor 1
DS-RGCs	Directionally selective ganglion cells
ED	Embryonic day
EHP	Elevated hydrostatic pressure
EPO	Erythropoietin
ERG	Electroretinography
GCL	Ganglion cell layer
GDNF	Glial cell-line derived neurotrophic factor
GLAST	Glutamate/aspartate transporter
GPx	Glutathione peroxidase
GSH	Glutathione
IGF-1	Insulin-like growth factor-1
IL-1β	Interleukin-1β
IL-6	Interleukin-6
INL	Inner nuclear layer
iNOS	Inducible nitric oxide synthase
IOP	Intraocular pressure
ipRGCs	Intrinsically photosensitive melanopsin-containing RGCs
J-RGCs	JamB expressing RGCs
KLF	Krüppel-like family
MAG	Myelin-associated glycoprotein
MHC-II	Major histocompatibility complex class II
mTOR	Mammalian target of rapamycin
ND4	NADH dehydrogenase subunit 4
NFL	Nerve fiber layer
NGF	Nerve growth factor
NgR	Nogo receptor
NLRP3	NOD-, LRR- and pyrin domain-containing protein 3
NMDA	N-methyl-D-aspartate
NO	Nitric oxide
NOS	Nitric oxide synthase
NRTN	Neurturin
NT-3	Neurotrophin-3
NT-4/5	Neurotrophin-4/5
OCT	Optical coherence tomography
OHT	Ocular hypertension
ONC	Optic nerve crush
ONH	Optic nerve head
ONL	Outer nuclear layer
PEDF	Pigment epithelium derived factor
PI3K	Phosphoinositide 3-kinases
PND	Postnatal day
PNS	Peripheral nervous system
PSPN	Persephin
PTEN	Phosphatase and tensin homologue
RAGE	Receptors for advanced glycation end-products
RGCs	Retinal ganglion cells
rhNGF	Recombinant human nerve growth factor
ROS	Reactive oxygen species
Sema3A	Semaphorin-3A
Sema5A	Semaphorin-5A
SOD	Superoxide dismutase
TLRs	Toll-like receptors
TNF	Tumour necrosis factor
TrK	Tyrosine kinase
TSPO	Translocator protein
VEGF-A	Vascular endothelial growth factor A
Zn^2+^	Zinc
αRGCs	alpha retinal ganglion cells
βRGCs	beta retinal ganglion cells
